# The Role of Copper in Alzheimer’s Disease Etiopathogenesis: An Updated Systematic Review

**DOI:** 10.3390/toxics12100755

**Published:** 2024-10-17

**Authors:** Angela Sabalic, Veronica Mei, Giuliana Solinas, Roberto Madeddu

**Affiliations:** 1 Department of Biomedical Sciences-Histology, University of Sassari, 07100 Sassari, Italy; rmadeddu@uniss.it; 2 Division of Thoracic Surgery, European Institute of Oncology (IEO), Scientific Institute for Research, Hospitalization and Healthcare (IRCCS), 20141 Milan, Italy; 3 Department of Biomedical Sciences-Biostatistics, University of Sassari, 07100 Sassari, Italy; mgsolinas1@uniss.it; 4 National Institute of Biostructure and Biosystem (I.N.B.B.), 00136 Rome, Italy

**Keywords:** Alzheimer’s disease, copper, ceruloplasmin, neurotoxicity, metals

## Abstract

Background: Alzheimer’s disease (AD) is the most common cause of dementia and cognitive decline in the elderly. Although the etiology of AD is unknow, an increase in amyloid precursor protein (APP) leads to the toxic aggregation of Aβ plaques. Several factors, such as hypertension, diabetes, dyslipidemia, smoking, hormonal changes, and metal exposure, could increase the risk of developing AD. In this review, we will examine the role of copper (Cu) in the pathophysiology of AD, as well as the mechanisms involved in neurotoxicity and cognitive decline. Methods: This review was conducted in accordance with PRISMA guidelines. We performed a comprehensive literature analysis over the last ten years on AD and Cu. Only late-onset Alzheimer’s disease was considered; only studies on elderly people of both sexes were included. Results: A total of seven articles were picked for this review, three studies focused on non-ceruloplasmin-bound Copper (non-Cp-Cu) and four on ceruloplasmin-bound Copper (Cp-Cu). The results showed higher Cu concentrations in patients compared to healthy controls. Conclusions: Elevated concentrations of Cu may contribute to the progression of AD, potentially interacting with ATP7B mutations, oxidative stress (OS), and amyloid-β plaques. Future research is needed to provide more robust evidence and better characterize the relationship between AD and Cu.

## 1. Introduction

Alzheimer’s disease (AD) is a progressive age-related neurodegeneration that represents the most common cause of dementia and cognitive decline in the elderly [1]. According to statistics, the true prevalence is unknown; however, it is the leading cause of death in the UK, surpassing cardiovascular diseases [2], and the fifth leading cause in individuals aged > 65 in the United States [3]. Additionally, it is twice as common in women compared to men [4]. Unfortunately, AD leads to a high level of health loss and mortality worldwide, with limited treatment options. Approximately 10–15% of AD patients are misdiagnosed by specialists, and the diagnosis can only be confirmed post-mortem. Early symptoms include cognitive deterioration, memory loss, and confusion.

The etiology of Alzheimer’s remains unknown; however, most cases present sporadically with late onset (≥65 years), while 5–10% of cases have a familial component, characterized by early onset (<65 years) and commonly associated with specific genetic mutations. The neuropathogenesis of AD is marked by the misfolding and aggregation of two proteins, amyloid precursor protein (APP) and tau protein (responsible for microtubule stabilization) [5]. APP leads to the formation of Aβ monomers, which aggregate to form Aβ fibrils and plaques, which are particularly toxic and damaging. On the other hand, hyperphosphorylation of tau protein leads to the formation of neurofibrillary tangles (NFTs) [6], associated with cognitive decline and the neurodegeneration typical of AD. Other genetic factors contributing to neurodegeneration are related to apolipoprotein E (apoE), which influences cytoskeletal integrity and neuronal repair efficiency. Studies have shown that other factors, such as hypertension, diabetes, dyslipidemia, smoking, hormonal changes, and metal exposure, can increase the risk of developing AD [6].

Metals are natural components of the Earth’s crust and can be inhaled or ingested through food, water, and air. Some heavy metals such as copper (Cu), zinc (Zn) and selenium (Se) can be detected in the body at low concentrations as trace metals. Metal ion homeostasis is essential in regulating certain brain functions. However, variations in concentration and form can make them toxic to the body. In particular, metals like Cu can promote the maturation of Aβ monomer aggregation and tau protein hyperphosphorylation. Evidence suggests that dysregulation of essential metal homeostasis and exposure to non-essential metals significantly impact AD pathogenesis [7].

Copper is a redox-active essential metal normally found in blood [8]. It is essential for the functioning of numerous enzymes, such as cytochrome C oxidase (CcO), copper-zinc superoxide dismutase (SOD1), dopamine-β-hydroxylase, tyrosinase, and lysyl oxidase, which respectively regulate the processes of energy metabolism, antioxidant activity, dopamine synthesis, melanin production, and tissue formation [9]. Cu is an important catalyst for iron absorption and heme synthesis. It is the third-most abundant transition metal in the liver, after iron (Fe) and zinc [3]. In the brain, Cu concentrations of approximately 60~110 μM can be detected, especially in the frontal lobe, brain, and hippocampus [8]. Usually, about 85–95% of total copper is found bound to ceruloplasmin (CP), a blue-looking copper glycoprotein. CP is mainly synthesized by the liver, brain, kidney, and fat. It plays important roles in Cu transport, Fe regulation, and antioxidant processes. Its structure is composed of 6 domains that can bind to six Cu atoms; when all six domains are bound to Cu, CP becomes unstable [10].

The remaining 5–15% of Cu is represented by loosely bound Cu to albumin and other little molecules, also known as “free copper” or “non-Cp-Cu”. Free copper, due to its loose binding, is freely available to meet tissue needs in the body.

The moment this 5–15% pool expands, copper becomes toxic; this is in line with what occurs in the case of Wilson’s disease [11]. Moreover, Cu is considered to be a pro-oxidant factor [12]: an increase in its serum levels may lead to feeding of the brain’s copper reservoir, which can enter oxidative stress (OS) cycles [12,13]. Neuronal damage caused by Cu homeostasis failure could be attributed to its numerous roles in processes essential for normal brain function, including catecholamine synthesis, activation of neuropeptides and hormones, antioxidant defense, connective tissue production, immune function, and synaptic transmission.

Considering the strong evidence of copper’s essential roles in the brain, it is not surprising that several studies have proposed that an imbalance in its homeostasis is associated with neurodegenerative disorders such as AD.

In this review, we will examine the role of copper in the pathophysiology of AD, as well as the mechanisms involved in neurotoxicity and cognitive decline, through a careful synthesis of literature published in the last 10 years, to identify new evidence.

## 2. Materials and Methods

### 2.1. Protocol

A draft protocol was written according to the Cochrane Handbook for Systematic Reviews of Interventions [14] for an updated systematic review on the role of the Cu in AD etiopathogenesis. The protocol is presented in the Supplementary Materials (S1).

### 2.2. Eligibility Criteria, Search Method, Information Sources, and Study Selection

The study adopted inclusion and exclusion criteria. These criteria were designed to ensure the selection of relevant studies while excluding those that did not meet the specified criteria.

The search was conducted in PubMed, Google Scholar, and across all databases available to us. We included only original research articles in full text, those published in a peer-reviewed journal, those in English and Italian languages, and those published from January 2013 up to January 2023. All case–control studies that addressed the analysis of Cu and AD were considered, following the combinations of keywords: “Alzheimer disease”, “blood”, “copper”, “risk”. We did not use any AND or OR in our research, thanks to the use of Boolean operators that read the space between words as an implied AND.

We considered only late onset Alzheimer’s disease; so, the population consisted in only over 60 years old people. Both sexes were taken into account. The nationality in which the study was conducted and/or where the samples were from was not a cause of exclusion.

After the initial screening of the title and abstract, the full texts of potentially eligible studies were examined independently by the authors and the discrepancies at all stages of study selection were resolved through discussion and consensus among the author group.

**Figure 1:**
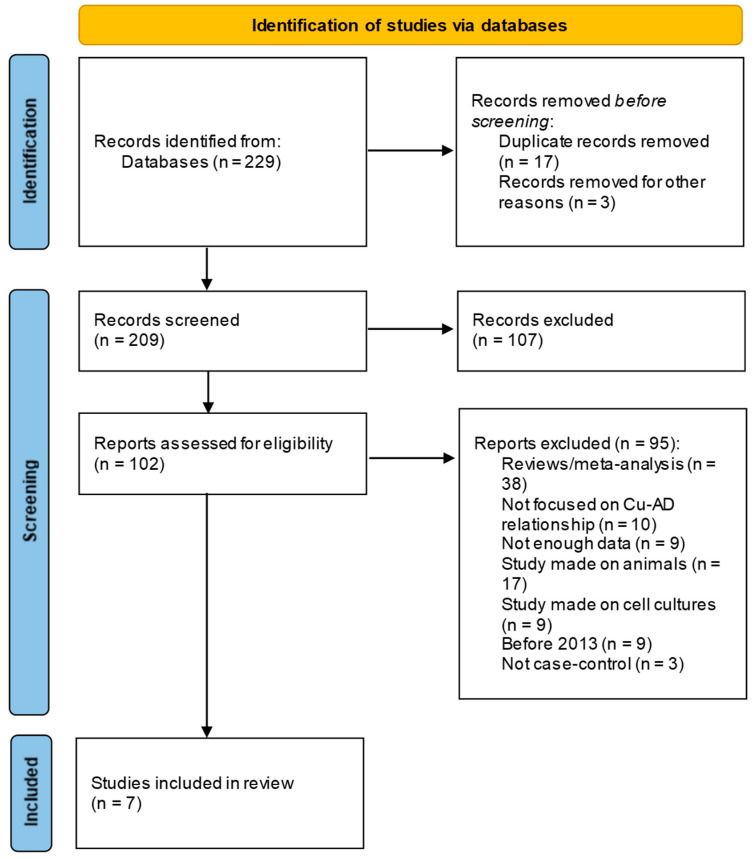
PRISMA flow diagram depicting the reasons for exclusion studies.

Finally, the studies included in the review, accompanied with the reasons for exclusion, are presented in the flow Preferred Reporting Items for Systematic Review and Meta-Analysis Protocols (PRISMA) [15] diagram (Figure 1). For data extraction, a standardized form is used (https://www.ncbi.nlm.nih.gov/books/NBK355732/ accessed on 17 November 2023) and screening of the data was performed using Excel spreadsheets.

## 3. Results

As reported in the PRISMA flow diagram (Figure 1), the search results from the electronic databases yielded 229 records; titles and abstracts of 209 total records were accessed in the screening phase, from which only 102 were selected for eligibility criteria. Finally, only seven studies (Table 1) were considered eligible for inclusion for full text-level review. A summary table of the characteristics of the seven records included in the review, with authors, year of publication, area of the study, age, gender number, levels of non-Cp-Cu (when stated) and Cp-Cu, for cases and controls, with the *p*-value emphasizing the difference between the two Cu types, is reported (Table 1). Out of the seven articles that were picked for this review, three studies focused on non-Cp-Cu and four on Cp-Cu. As follows, a narrative synthesis of the articles included in the review was undertaken for non-Cp-Cu and Cp-Cu, respectively.

In all studies, fasting blood samples were collected in the morning, and serum was rapidly stored at −80 °C. The Cu concentration was measured using inductively coupled plasma optical emission spectroscopy (ICP-OES) or atomic absorption spectrophotometry.

### 3.1. Ceruloplasmin-Bound Copper Results

Ceruloplasmin-bound copper represents most of the pool of blood Cu. In this section, we present the four studies of choice on this topic. Al-khateeb et al. [12] studied the association between serum copper, lipids concentration, and changes in cognitive function in elder Jordanian subjects (52 dementia patients and 50 controls). The results showed no significant difference between the dementia and control groups for age (*p* = 0.215) and gender (*p* = 0.290), respectively, while educational level (*p* = 0.006) and coffee intake (*p* = 0.000) showed significant associations. Al-khateeb et al. stated that caffeine is normally present in Jordanians’ life through daily coffee intake. They theorized that caffeine, as a stimulant that mimics adenosine, could be used by some neurons, stimulating neuronal activity; this neuronal activity was then speculated to offer protection against dementia. A study tested this hypothesis on a mouse model by administrating pure and crude caffeine, showing that crude caffeine could suppress Aβ in mice with AD [12].

In this study, serum Cu in both groups was within the normal physiological range (114.55 ± 57.6 µg/dL in patients vs. 126.12 ± 71.78 µg/dL in controls). Finally, the correlation analysis did not show a correlation between mental scale score (MMSE) and serum total cholesterol (TC), triglyceride (TG), high density lipoprotein HDL, low density lipoprotein LDL, and Cu levels in the dementia group, except the correlation for age (*p* = 0.005).

In 2021, Yadav J et al. and colleagues [19] analyzed the correlation between different essential and non-essential metals concentration, such as arsenic (As), lead (Pb), cadmium (Cd), mercury (Hg), aluminum (Al), Zn, Fe, and Cu and the expression of four genes, *APP*, *PSEN1*, *PSEN2*, and *APOE4*, in Alzheimer’s patients (n = 50) with age-matched control subjects (n = 50). The results showed higher levels of Cd and Hg (*p*-value < 0.0001) and Cu compared to Fe and Zn, which were found in lower concentrations in AD patients. On the other hand, although apolipoprotein E (Apo E) has been associated with the amyloid-β (Aβ) pathology involved in AD and showed a maximum affinity with copper, in this study, the correlation between metal concentration and gene expression was not found to be significant in the AD cases.

Negahdar H et al. [16] enrolled 120 elderly patients with cognitive impairment (MCI) and 120 elderly healthy people who were differentiated using MMSE in order to determinate serum levels of Cu, Zn, manganese (Mn), and homocysteine (Hcy) in both groups. This study also analyzed the levels of malondialdehyde (MDA), a product of lipid peroxidase used to analyze the level of OS, through the thiobarbituric acid reactive substance (TBARS) assay; further analyses to evaluate the antioxidant power were performed through the use of the ferric reduction antioxidant power (FRAP) assay. Additionally, the authors hypothesized an antioxidant role for Aβ, based on its concentration. The TBARS and FRAP assay results showed lower concentrations in patients than in healthy controls (*p* < 0.001).

Moreover, the study showed the decrease in gradient from mild to severe cognitive impairment patients (MCI I to MCI II). There was no significant difference in trace elements between groups and Hcy levels for age, gender, and education. Thus, the results of this study confirmed the association between oxidative status and increase in the severity of cognitive impairment.

Paglia et al. [17] proposed a study aimed to profile serum biomarkers during the progression of AD. The study involved 40 healthy subjects, 24 subjects with subjective memory complaint (SMC), 20 MCI subjects, and 20 AD patients. They analyzed 22 serum elements, such as Mn, Fe, Cu, Zn, Se, Hg, thallium (TI), antimony (Sb), vanadium (V), and molybdenum (Mo). All of these elements showed significant changes in the four groups examined. Levels of several essential elements, such as manganese, selenium, zinc, and iron, tended to be increased in SMC and progressively decreased in MCI and AD. Toxic elements showed a variable behavior, since levels of some elements tended to increase, while levels of others tend to decrease in AD.

### 3.2. Non-Ceruloplasmin-Bound Copper (Non-Cp-Cu) Results

For several years, non-Cp-Cu was considered a topic only regarding Wilson’s disease (WD), but recently it has been reported that there might be a correlation with Alzheimer’s disease as well. An interesting study focused on non-Cp-Cu was conducted by Squitti R. et al. [13]. The study showed homogeneity in the selection of their patients: they were in a range of 72–80 years old, with mostly female participants. Where the age between cases and controls was over 9 years, it was corrected through the use of statistical means. The study showed a correlation between the increase in non-Cp-Cu and the risk of AD.

Squitti R. et al. (2013) [13] investigated the role of the *ATP7B* gene in copper dyshomeostasis associated with AD, measuring serum levels of ceruloplasmin, copper, and free copper, and evaluating their association with certain single nucleotide polymorphisms (SNPs)—such as rs1801243, rs2147363, rs1061472, and rs732774—of the ATP7B gene. They stratified the population into “low free copper” (<1 µmol/L), “medium free copper” (≥1, <1.6 µmol/L), and “high free copper” (≥1.6 µmol/L). Analyses of demographic, clinical, and molecular characteristics showed that AD patients and healthy controls differed in terms of age, APOE genotype, and MMSE score. The age-adjusted analysis revealed that free copper and copper levels were higher compared to controls (*p* < 0.001), while ceruloplasmin levels did not differ. The *ATP7B* gene variants were analyzed only in the group with high serum copper levels, revealing that all SNPs showed no differences between the two groups and were in Hardy–Weinberg equilibrium. AD individuals homozygous GG for rs7323774 had increased free copper levels in serum. Additionally, in 2017 [11], the same authors analyzed SNPs in a total of 287 AD patients, divided into two groups based on differences in their non-Cp-Cu levels using a cut-off of 1.9 μM. The results showed that SNPs rs1061472 A>G (exon 10) and rs732774 C>T (exon 12) of *ATP7B* differed in AD patients in relation to the established cutoff. Overall, 60% of the patients evaluated in the study had non-Cp-Cu values above normal.

Squitti R. et al. (2017) [18] observed a total of 56 diabetic patients compared to 28 healthy controls. It demonstrated that type 2 diabetes (T2D) patients have a higher risk of contracting AD. In fact, AD and T2D patients had very similar values of non-Cp-Cu (2.1 µmol for AD and 2.0 µmol for T2D), way higher than controls. The cause of this copper dysregulation might be attributed to the fact that both AD and T2D share a common pathway.

## 4. Discussion

AD is a neurodegenerative condition that causes permanent damage to several processes, such as thinking, memory, and linguistic ability. AD is the most prevalent type of dementia among elderly patients (>65 years old). The main risk factors for AD are the interaction between environmental factors, genetic variants, lifestyle (abuse of alcohol, smoking, nutrition, obesity, and sedentariness), immune system dysfunctions, inflammations, and oxidative stress from metal ions. Cu toxicosis, or its accumulation, has mostly been disregarded up until now because while in WD it causes motor problems, in AD it produces cognitive problems [13].

Squitti et al. analyzed the correlation between ATP7B and Cu in patients with AD in order to investigate the role of ATP7B in Cu dyshomeostasis. Specifically, in hepatocytes, ATP7B donates Cu to ceruloplasmin. This study showed that AD patients, GG homozygous for ATP7B rs7323774 SNP, had higher serum non-Cp Cu concentrations than healthy controls [13]. These data led to the conclusion that gene variants of ATP7B could trigger an increase in free Cu levels in serum, activating APP and increasing the susceptibility to developing AD [20]. Therefore, the authors hypothesized that ATP7B, like APOE, could represent a genetic risk factor for the development of AD, although they share independent pathogenic pathways [13,18]. Recent research has suggested that the metallochaperone antioxidant-1 (Atox1) and protein copper metabolism MURR1 domain-containing (COMMD1) genes could play a role in copper dyshomeostasis breakdown and could also impact the clinical phenotype and age of onset of the disease [21,22]. COMM1 in dogs, particularly in Bedlington terriers, causes canine copper toxicosis with high hepatic copper accumulation, which is responsible for hepatitis and cirrhosis and, if untreated, leads to the animal’s death. In the literature, it is reported that human COMMD1 encodes a protein that is 88% identical to its canine homolog. Moreover, COMMD1 interacts with the N-terminal domain of ATP7B. The similarity of symptoms between canine copper toxicosis and Wilson’s disease (WD) with atypical phenotypes has led to the idea of a possible role of COMMD1 in patients with WD [21]. These loci in the ATP7B, ATOX1, and COMMD1 genes are still poorly understood; however they may have a significant impact on the probability of developing copper-associated AD [13]. Moreover, variants in the ATP7B gene [21,22] may play a key role both in sabotaging Cu homeostasis and in the age of onset of AD. It also appears that Cu dysfunction caused by ATP7B is a causative rather than associative risk factor for AD [13,18]. It is worth noting that most studies on ATP7B gene variants have been conducted primarily on the Italian population, while only the study by Liu H. et al. analyzed three missense SNPs (rs1801243, rs1801244, and rs1801249) in the ATP7B gene in the Taiwanese population, suggesting that genetic variations in the copper transporter gene ATP7B might contribute to AD in this population. It could be hypothesized that there is genetic variability related to ethnicity; therefore, further studies are needed to better understand the role of the ATP7B gene and its variability in ethnic groups [23]. Interestingly, these genes also interact with other essential ions in the body, such as Fe, Zn, Mn, Mg, and Se. Yadav et al. found higher levels of Cu and lower levels of both Zn and Fe in serum; this has been further confirmed in other studies. Hozumi et al. reported increased levels of both Cu and Zn in cerebrospinal fluid and the cortex while also reporting lower levels of Zn in serum [24]. Brewer et al. further confirmed higher Zn concentrations in the brain and lower concentrations in serum, suggesting that its deficiency could be due to a wrong diet [25]. Damante et al. reported that both Zn and Cu compete for the same binding sites in Aβ. While Cu bound to Aβ becomes more toxic, Zn promotes the production of fibrils [26]. Another study [27] hypothesized that both of the aforementioned elements could participate in AD pathogenesis. More specifically, dyshomeostasis of both Zn and Cu could induce the formation of Aβ aggregates, which can become amyloid fibrils of said elements. Aβ monomers can then produce Aβ plaques when associating with fibrils through an autocatalytic fibrillation of Aβ triggered by the fibrils. Cu ions can become integrated into the plaques, becoming unavailable for protein-binding metals and thus causing OS. Some metals can travel past the blood–brain barrier (BBB), where they produce free radicals and cause oxidative stress, as well as mitochondrial and neuronal injury. Also, post-mortem studies of brain tissues in AD patients have shown the presence of high concentrations of Cu, Zn, and Fe around Aβ plaques. On the contrary, Mg in the brain is vital for BBB integrity and function; additionally, high levels of Mg reduce BBB permeability [28]. A dysfunction in the BBB might reduce Mg concentrations, the latter being an event that has been seen in multiple AD patients [29].

Negahdar et al. hypothesized that under oxidative stress conditions, amyloid-β plaques exert a chelating role toward Cu as a compensatory action. A reduction in serum Cu concentration correlated with the severity of AD was observed; patients with MCI II had higher Cu concentrations compared to patients with MCI I and healthy subjects [30]. Conversely, other studies have observed a reduction in serum Zn and Fe concentrations and an increase in Cu concentration [17,19,31,32]. Moreover, Yadav et al. suggested that more metals are involved in AD, such as Cd, Al, and Hg. None of these elements had a significative correlation with gene expression in AD. More specifically, Hg concentrations were found to be higher in isolated subcellular fractions of the brain [33] and Al concentrations in the hippocampus and the entorhinal cortex region of the brain. Specifically, Al was found to be another cause of OS by altering cell membranes and enhancing iron lipid peroxidation, forming H_2_O_2_ and OH radicals [34] and causing neuronal and glial death [35]. Cd, alongside Pb and As, seems to be responsible for altering the gut microbiome, leading to the formation of toxic bacterial species populations [36]. This alteration seems to be connected to AD [37]. As upregulates many inflammatory signaling molecules, such as tumor necrosis factor-α, interleukins, and NF-κB, causing further oxidative stress [38]. Moreover, Pollack et al. and Wang et al. found that higher concentrations of Pb [39] and Cr [40] would increase Hcy levels. Higher levels of Hcy have been associated with greater risks of dementia and cognitive impairment [41] as well as cardiovascular events [42].

APOE protein has also been shown to have a particular affinity for Cu and less for Zn. Elevated Cu concentrations are also detectable in other cognitive disorders, such as MCI and SMC [17], as well as in other conditions like type 2 diabetes (T2D) and dyslipidemia. Tissue damage caused by hyperglycemia leads to the impairment of copper homeostasis. As demonstrated by numerous authors, patients with T2D have high serum levels of both total Cu and non-Cp Cu, a situation also observable in the serum of AD patients [18,43]. Furthermore, the formation of Aβ chains induced by OS has been detected in the pancreatic tissues of patients with T2D [18]. Evidence suggests that AD and diabetes share common pathways. Moreover, it has been observed that the multi-ligand receptors AGEs are responsible for various inflammatory processes by transporting circulating Aβ chains that induce OS common to diabetes, obesity, and AD [44,45]. Studies with murine models have shown that Cu stimulates the deposition of Aβ plaques in the brains of mice fed cholesterol [46]. Although the role of cholesterol in cognitive decline is still unclear, differences in the lipid profiles of AD patients and those at high cardiac risk have been observed, suggesting a correlation between atherosclerosis and AD, with high serum cholesterol concentrations as a common risk factor [47], implying that a condition of hypercholesterolemia in young adults could influence the onset of AD in later life [48]. However, there is not enough evidence to define the lipid profile as a risk factor for AD; probably, multiple factors, including vascular damage caused by hyperlipidemia, contribute to neurovegetative disorders. At the same time, in vivo laboratory studies aiming to vary the diet have found an inverse relationship between copper and cholesterol, suggesting that cholesterol reduces hepatic copper concentrations. A study conducted by Singh et al. [49] in vivo and in vitro found that small amounts of copper ingested through water could reach the brain via the bloodstream, causing OS and contributing to the malfunction of protein 1, which is responsible for clearing Aβ plaques from the brain. However, Al-Khateeb et al. [12] analyzed Cu concentrations in the water and serum of patients involved in the study, finding that Cu levels in water did not significantly impact serum Cu levels. The same authors also found significant differences between patients with cognitive impairment and control subjects regarding the level of education. This result was also confirmed by Stern et al., who studied a cohort of 58 elderly people with different educational backgrounds. The authors showed that education did not develop immunity from AD but definitely slowed its development. They assumed that education could reflect cognitive capacity and that it could provide a set of cognitive tools that would let the patient compensate for the changes that occur during AD progression [50].

All of these observations highlight that genetic changes, lifestyle, and exposure have different effects on copper metabolism and its involvement in AD. However, it is complex to define a physiological range of Cu as its concentrations can vary from individual to individual based on age and health status. It is evident that in the various studies analyzed in this review, serum Cu concentrations were higher in AD patients compared to controls.

To our knowledge, this work is the first review conducted to provide updated evidence from the literature since 2013 on the role of the Cu in the pathophysiology of AD, as well as the mechanisms involved in neurotoxicity and cognitive decline. However, the updated literature search conducted in this review has its limitations. Firstly, the literature search was focused only on case–control studies on the serum of AD patients; therefore, additional relevant studies might have been missed. Secondly, we excluded articles published in preprint databases due to the lack of peer review.

## 5. Conclusions

This review summarizes the updated 10-year findings on the relationship between AD and Cu, which may provide a new perspective and direction for future scientific research; preventive measures against AD, such as changes in lifestyle and/or diet; as well as development of new therapies.

Future research is required to provide more robust evidence to characterize the relationship between AD and Cu as well as the balance and interaction between different metal ions in the etiopathogenesis of disease.

## Figures and Tables

**Table 1:** Characteristics of seven included studies.

Authors (Publication Year)	Country	Cases				Controls			
		Cu Type	Gender Number	Age (Mean ± Sd)	Cu (Mean ± Sd)	Subject Gender	Age (Mean ± Sd)	Cu (Mean ± Sd)	*p*-Value (Cu)
Al-khateeb E. et al. (2014) [12]	Jordan	Cu	23F/29M	70.7 ± 7.63	126.12 ± 71.78 (μg/dL)	17F/33M	68.9 ± 7.11	114.55 ± 57.6 (μg/dL)	<0.377
Negahdar H. et al. (2015) [16]	Iran	Cu	60F/60M	74.2 ± 6.9	0.99 ± 0.4 (ppm)	60M/60F	67.7 ± 7.3	0.88 ± 0.3 (ppm)	<0.068
Paglia G. et al. (2016) [17]	Italy	Cu	25F/9M	72.4 ± 7.48	815.75 ± 206 (μg/L)	25F/15M	65.53 ± 6.37	703.88 ± 244.03 (μg/L)	<0.033
Squitti R. et al. (2013) [13]	Italy	Non-cp Cu	294F/140M	74.9 ± 8.1	2.24 ± 3.14 (μmol/L)	207F/96M	66.5 ± 10.5	0.28 ± 2.98 (μmol/L)	<0.001
Squitti R. et al. (2017) [11]	Italy	Non-cp Cu	52F/37M	73 ± 8.5	2.3 ± 1.5 (μmol/L)	77F/70M	49 ± 12.7	1.07 ± 0.6 (μmol/L)	<0.001
Squitti R. et al. (2017) [18]	Italy	Non-cp Cu	118F/58M	80.7 ± 6.9	2.5 ± 0.5 (μmol/L)	76F/35M	81.5 ± 6.8	1.6 ± 0.3 (μmol/L)	<0.0001
Yadav J. et al. (2021) [19]	India	Cu	32F/28M	74.1 ± 1.68	0.127 ± 0.024 (mg/L)	-	74.13 ± 1.68	0.069 ± 0.0068 (mg/L)	<0.0254

## Data Availability

The original contributions presented in the study are included in the article/Supplementary Material, further inquiries can be directed to the corresponding author/s.

## Supplementary Materials

The following supporting information can be downloaded at: https://www.mdpi.com/article/10.3390/toxics12100755/s1, Text of the protocol S1: Our protocol based on the Cochrane Handbook for Systematic Reviews of Interventions.
