# Supplementary material for: The Role of Copper in Alzheimer’s Disease Etiopathogenesis: An Updated Systematic Review
Source: Toxics. 2024 Oct 17;12(10):755. doi: 10.3390/toxics12100755 (PMC11511397; doi:10.3390/toxics12100755)
Supplement: Supplementary file 1 [file toxics-12-00755-s001.zip › toxics-3236164-supplementary.pdf]

PROTOCOL

Review Title: THE ROLE OF COPPER IN ALZHEIMER'S DISEASE ETIOPATHOGENESIS: AN UPDATED SYSTEMATIC REVIEW.

Review Question. Does an increase in copper in the blood also increase the risk of developing Alzheimer's disease?

Identification of studies. The review follow the PICO framework: (Patient or Population, type of Interventions (and Comparisons if there is any), and the type of Outcomes that are of interest). The most recent studies possible will be used (last 10 years), regardless of the location where they were conducted. Study quality will not be a reason for exclusion, except in the case of a too small sample size, which would be statistically insufficient. Studies must have been conducted on groups of people, not animals, for greater accuracy. Individuals considered must be over 60 years old, as young-onset Alzheimer's (30-60 years) will not be considered. Participants can be both men and women.

Search strategy. The databases used for the research will be PubMed and Google Scholar and all databases available to us. Unpublished statistics will not be used as they are difficult to obtain; also it would be challenging to trace their sources and verify their accuracy. Studies considered will be in both English and Italian for easier comprehension. The following combinations of keywords relating to Alzheimer disease and copper were used: “Alzheimer disease”, “blood”, “copper” and “risk”. We didn’t use any AND or OR in our research, thanks to the usage of the Boolean operators which read the space between words as an implied AND.

Type of studies to be included. Only the original research articles in full text will be included, published in a peer-reviewed journal, in English and Italian languages and published from January 2013 up to January 2023. All case-control studies that addressed the analysis of copper and Alzheimer’s disease will be considered.

Data extraction. The data will be extracted using a standardized form (https://www.ncbi.nlm.nih.gov/books/NBK355732/). The information will include: author, year, country, characteristics of participants, details of interventions, measures of outcomes, main results. Finally, data will be summarized in a specific table.

Summary from included studies. The synthesis of studies selected at the end of the process will be presented narratively.

Ethical considerations. Specific ethical considerations are not necessary for this review, as it does not involve direct interaction with patients.

Dissemination. The results of the review will be published in a peer-reviewed journal and presented at national and international meetings.
